# The Changes in Electrical and Interfacial Properties of Polyimide Exposed to Dielectric Barrier Discharge in SF_**6**_ Medium

**DOI:** 10.1155/2013/890454

**Published:** 2013-06-10

**Authors:** Hafiz Z. Alisoy, Murat Koseoglu

**Affiliations:** Department of Electrical-Electronics Engineering, Inonu University, 44280 Malatya, Turkey

## Abstract

The formation mechanism of space charges in polyimide (PI) which was exposed to dielectric barrier discharge (DBD) in SF_6_ medium and the effects of the space charges on interfacial and electrical properties of PI were investigated. The variation of normalized surface charge density on PI sample was calculated and illustrated for different DBD exposure times. The surface potential was measured to determine the effect of the space charges on the sample. Then, the contact angle values were measured to obtain the relation between the surface energy and the surface charge density. The expressions for the total charge and the concentration of trapped electrons were derived by using Poisson and continuity equations at stationary state. The space charges were determined experimentally by using thermally stimulated depolarization current (TSDC) method. Also, SEM image and FTIR spectrum of virgin and treated samples were presented to observe the structural variations. It was seen that the approach for the formation mechanism of the space charges agreed with the experimental data. However, it was concluded particularly for the short-time DBD treatments that the space charges accumulated in the sample should be considered besides the effects of surface functionalization in the determination of the surface energy.

## 1. Introduction

Polyimides (PI) are widely used as insulating, substrate, or interfacial material in several industrial applications such as mechanical, electrical, medical, and aeronautics, since they have excellent mechanical, electrical, thermal, and chemical properties. Any change in electrophysical properties of the material resulting from the exploitation conditions can have considerable negative effects on the operational utilization of the material. So it is very important to determine the changes in the electrophysical properties of the material under different exploitation conditions (strong electrical fields, partial discharge, etc.). On the other hand, these layered and molded materials include gas voids because of technological reasons. A partial discharge, which is occurred in the structure because of any reason during the exploitation of dielectric, affects the material considerably and can cause different failures [1–5]. As known, the partial discharges have important destructive effects on the material such as electron-ion bombardment, radiation, and thermal. So these complex factors must be considered in the exploitation of polymer dielectrics as insulating or substrate material in the industrial fields. Recently, new polymeric materials, which have better thermal, electrical, and mechanical properties, have been produced due to the advanced production technologies. Moreover, the properties of the materials can also be improved by different physicochemical treatment methods such as corona discharge, glow discharge, and dielectric barrier discharge (DBD). DBDs are very attractive plasma treatment methods for improvement of adhesion properties [6]. 

DBD is a highly transient, low-temperature, nonequilibrium discharge formed from electrons of high mean energy which exists in a broad range of pressures. During the development of DBD, the charge carriers collect on the dielectric; they reduce the field strength in the discharge area, and eventually cause the discharge to quench. The DBD proceeds in most gases through a large number of separate current filaments referred to as microdischarges. These microdischarges have complex dynamic structure and are formed by channel streamers that repeatedly strike at the same place as the polarity of the applied voltage changes. The persistence of streamers to strike at the same place of their ancestor results from the memory effect. The memory effect is associated with charge deposited on the dielectric barrier, as well as on residual charges and excited species in the microdischarge channel [7–10].

High-energy electrons are effective in the generation of activated species, functional groups (NH, OH), and radicals on the surface. These formations on the surface of the sample are considerable factors on the electro-physical properties of the materials [6–8, 11, 12]. The influence of DBD treatment on the surface of polyimide (PI) results in important changes in the surface energy of the sample [11, 13–15]. However, the concentration of polar groups formed on the surface reaches saturation quickly. But the decrease in contact angle and discharge current due to the exposure time cannot be explained by only the formation of polar groups and functionalities. In addition to these, the concentration of free radicals measured by EPR (electron paramagnetic resonance) method is not sufficient to elucidate the increase in the polar component of surface energy. It is supposed that the effect of space charge formation on the surface energy of the material should not be neglected under definite conditions. It was seen that plasma treatment of PI results in the formation of space charges in the material due to the diffusion of charge carriers from plasma channel [5]. Therefore, it may be thought that there is a correlation between surface energy-*W* and surface charge density-*σ*, which is valid for the PIs having various structures under definite conditions [5, 12, 16]. 

 It has been determined in many investigations that the electrical strength, dielectric constant, dielectric loss angle, and conductivity change during aging process of polymer [2, 16, 17]. It is not enough to elucidate the change of these properties only with the variation of surface properties. So in order to explain the changes mentioned above, the volumetric mechanism of aging must be analyzed well. The effects of space charges, which formed in polymer structure, on the electrical properties of polymer were investigated in [2, 18, 19]. The space charge formation was observed in almost all polymeric materials used in electrical and electronics technology. For this reason, the investigation of distribution characteristics of space charges formed in the structure of polymer exposed to electrical discharges is very important for theoretical and practical aspects of electronic and plasma applications [5, 8, 12, 18, 20]. 

 In this study, some experimental results related with the formation of space charges in PI dielectric by DBD effect in SF_6_ medium were presented. The relation between the surface charge density and the surface energy was presented in accordance with the measurements of surface potential and contact angle. Furthermore, the expressions for the total charge value in the samples and the concentration of trapped electrons were derived by using Poisson and continuity of the current equations at stationary state. We think that the obtained results are important for the failure analysis of the material which contains space charges resulting from partial discharges. 

## 2. Experimental

Firstly, the prepared samples were exposed to DBD in SF_6_ medium for different times, and then the surface potential, the contact angle, and TSDC currents of the samples were measured to calculate the surface charge density, to estimate the surface energy and to analyze the conduction mechanism of the sample, respectively. In addition, FTIR spectrums and SEM images of the treated and virgin samples were presented to analyze the structural behavior of the samples. 

The method to determine the space charge formation in the sample due to the DBD effect is similar to one given in [21, 22]. For this purpose, thin polyimide film PM-A (TU 6-19-121-85) whose international name of analogues is “Kapton” was used in this study. The properties of the samples were as follows: dielectric strength ~200 kV·mm^−1^, volume resistance ~10^18^ Ω^−1^·cm^−1^, dielectric permittivity ~3.5, loss factor (tan *D*) ~0.002 at 50 Hz, and density ~1.42 g·cm^−3^. The samples of PI material having average thickness value of 42 *μ*m were prepared as 5 cm × 5 cm sheets. The surfaces of the samples were cleaned with ethyl alcohol, and then in order to treat the samples thermally, they were placed between aluminum electrodes which were connected with a short-circuit wire for 30 min. The treatment temperature was chosen as 180°C. After thermal treatment, the samples were labeled as 1, 2, and 3. One side of each sample was coated with 1 cm diameter and 3-4 *μ*m thick of Al electrode in vacuum conditions. The sample, labeled as 1, was chosen as virgin sample. The sample, labeled as 2, was used to measure surface potential and contact angle. Surface energy was determined from Young-Dupre equation by contact angle goniometry method using distilled water [5]. The sample, labeled as 3, was used to measure TSDC currents. After these preparatory steps, the samples labeled as 2 and 3 were exposed to DBD in the time interval of 1 min−6 hours in the chamber where the coated sides of the samples were back to back.

**Figure 1:**
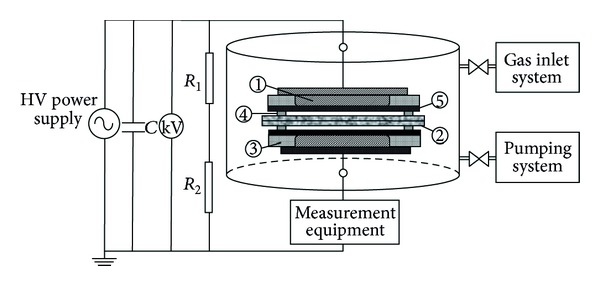
Experimental setup of the DBD treatment: (1) metal electrodes, (2) sample, (3) plexiglass rings, (4) gaskets, and (5) dielectric barrier.

A schematic diagram of the experimental setup, which was employed to activate the surface of the sample by DBD method, was presented in Figure 1. The metal electrodes (1) were made of stainless steel and embedded into the plexiglass rings (3). The gaskets (4) determine the width of the gas gap between the sample (2) and dielectric barriers (5) in discharge gap. Before the experiment, the DBD chamber was evacuated three times until the value of 1.33 × 10^−3^ mbar was reached. The voltage applied to the discharge cell was 14 kV, and the pressure in the chamber was 1 atm. During the DBD, the current was measured as 45 *μ*A. Ignition voltage of the discharge was determined by monitoring the impulse current on oscilloscope. After DBD treatment, the sample was taken out the DBD chamber. Then, the surface potential, contact angle, and TSDC spectrums were measured for the activated sample. 

**Figure 2:**
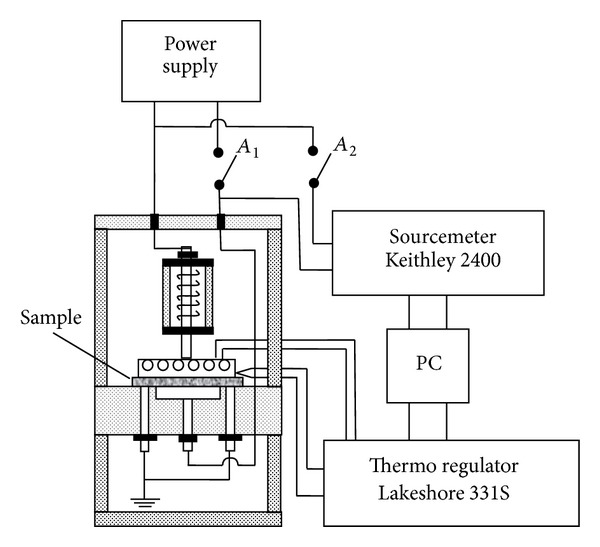
Schematic diagram of measurement system for TSDC.

The space charges accumulated in the sample due to the DBD were investigated by using the experimental setup shown in Figure 2. The surface charge density of the sample was measured by compensation potential method [2, 5, 12]. The maximum compensation potential was set at 3000 V, and minimum value was set at 25 V. Experimental error was about 10%. Initially, compensation potential-*V* was determined, and then (1) was used to calculate the surface charge density:

(1) $$\begin{array}{c} \sigma =\frac{\varepsilon_{0}\varepsilon_{r}}{d_{}}V, \end{array}$$

where *ε*_*r*_ is the dielectric constant of the sample, *ε*_0_ is the dielectric constant of vacuum, and *d* is the thickness of the sample. The sectional region affected by the space charges was quite shallow in comparison with the thickness of the sample. So the average value of *ε*_*r*_ changed negligibly small due to the short discharge time for the experimental conditions [23].

## 3. Results and Discussion 

One of the most important factors, besides polar groups and surface functionalization, influencing the electro-physical properties of the polymers exposed to DBD is the space charge formation in the material. The formation of space charges in polymers can be explained as follows. As known, polyimide has mesomorph structure, amorphous and crystalline regions. In crystalline regions of polyimide, energy spectrums of electrons are characterized by band structure. Trapping levels resulting from the disorders in the atomic structure are commonly at the bottom of the conduction band, and density of these levels decreases towards the inferior regions of forbidden gap. At first, these levels are not occupied. During DBD process, some electrons settle onto the surface of the sample from the discharge region, and some electrons emerge on the sample surface due to the dissociation of SF_6_^−^, SF_5_^−^, and F_2_^−^ ions on the surface. Then, the electrons formed on the surface pass to the conduction band of the sample [5, 18]. These electrons transfer their energies to the lattice, and they are captured by localized energy levels. In course of time, they pass through the lowest trapping level, and successive electrons occupy the trapping levels neighboring to the lowest trapping level. Therefore, the space charges can move in the bulk after the electrons occupy the most of trapping levels near the surface. The following behavior of space charges can be in two different forms: (i) tunneling of charges in trapping levels into the conduction band due to the thermal activation or (ii) hopping between trapping energy levels. These behaviors will result in the degradation of the insulating properties of the material, so the formation of breakdown and failure in the material become easier. In the investigated mechanism, it is not needed any electrical field for the injection of charges into the polymer. The density gradient of charges is sufficient to form space charges in the bulk.

**Figure 3:**
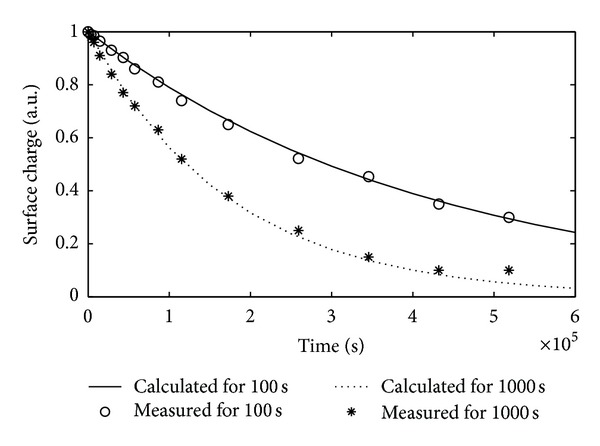
The variation of normalized surface charge density (*σ*/*σ*_*m*_) versus decay time for the sample exposed to DBD in SF_6_ for 100 s and 1000 s (*σ*_*m*_^100^ = 7.86 · 10^4^ nC/m^2^, *σ*_*m*_^1000^ = 17.47 · 10^4^ nC/m^2^).

 The initial values of surface charge density for PI samples exposed to DBD were determined by using (1) as 7.86 · 10^4^ nC/m^2^ and 17.47 · 10^4^ nC/m^2^ for the exposure times of 100 s and 1000 s, respectively. The change of relative surface charge density of PI samples after DBD treatment was illustrated versus decay time in isothermal conditions in Figure 3. As seen from the figure, the relative surface charge density decreased nonlinearly with time since the charge carriers seized in localized energy states became free. 

**Figure 4:**
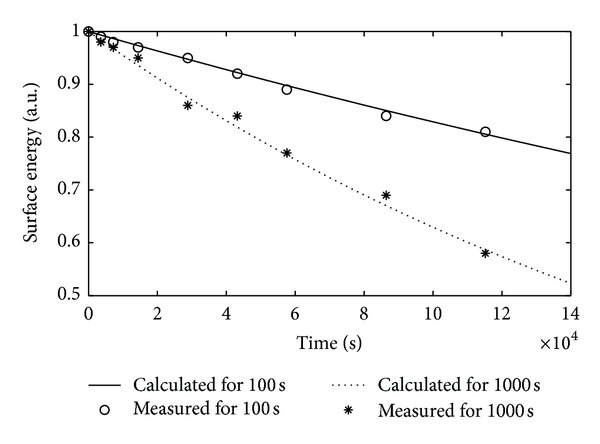
Normalized surface energy (*W*/*W*_*m*_) variation of PI exposed to DBD in SF_6_ medium for 100 s and 1000 s (*W*_*m*_^100^ = 0.122 J/m^2^, *W*_*m*_^1000^ = 0.141 J/m^2^).

**Figure 5:**
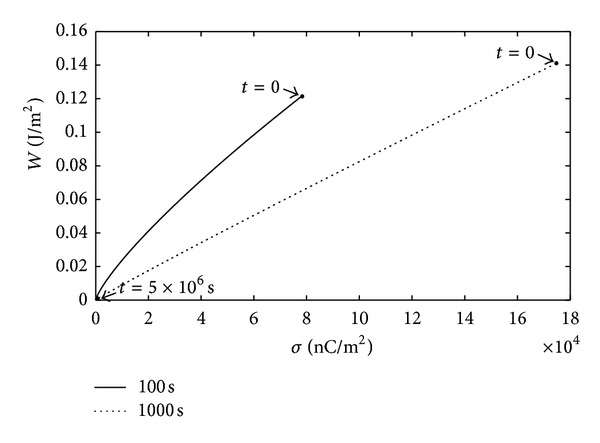
Variation of surface energy (*W*) versus surface charge density (*σ*) in the decay time of 0–5 · 10^6^ s for PI sample exposed to DBD in SF_6_ medium for 100 s and 1000 s (at *t* = 0, *W*_*m*_^100^ = 0.122 J/m^2^, *W*_*m*_^1000^ = 0.141 J/m^2^; *σ*_*m*_^100^ = 7.86 · 10^4^ nC/m^2^; *σ*_*m*_^1000^ = 17.47 · 10^4^ nC/m^2^).

The measured contact angle values were utilized to calculate surface energy values of the samples exposed to DBD in the durations of 100 s and 1000 s. The initial values of surface energy of the samples were calculated as *W*_*m*_^100^ = 0.122 J/m^2^ and *W*_*m*_^1000^ = 0.141 J/m^2^ by Young-Dupre equation, respectively. Young-Dupre equation is used to describe the interactions between the forces of cohesion and adhesion and measure what is referred to as surface energy [24]. It is very important to measure contact angle value in the estimation of the energy states of the molecules and the adhesive properties of the surface on the boundary of two phases. Young–Dupre equation used to estimate the work of adhesion *W* was given as follows [25]:

(2) $$\begin{array}{c} W=\gamma \left(1+\cos\;\theta \right), \end{array}$$

where *γ* is the surface tension of distilled water which has a value of 72.14 × 10^−3^ N/m^2^ at 20°C. The changes in relative surface energy of PI samples were shown versus decay time in isothermal conditions in Figure 4. As seen, the relative surface energy values of the samples decreased with time. Then the relation between surface energy and surface charge density was examined in accordance with the obtained results [5]. The illustration, which depicts how the surface energy changed versus the surface charge density after DBD treatment, was presented in the decay time of 5 · 10^6^ s in Figure 5. It was observed that the increase in surface charge density corresponded to an increase in surface energy, thus the decreases in surface energies of DBD treated samples due to the time can not be attributed to only the relaxation of activated species, functional groups (NH, OH), and radicals. The surface charge density should also be considered besides mentioned factors influencing the surface energy. 

**Figure 6:**
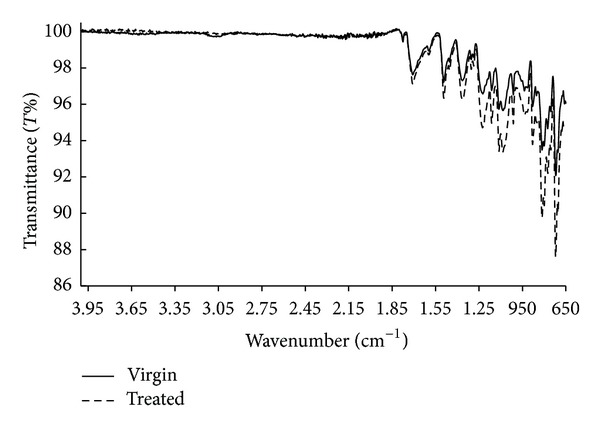
The FTIR spectrum of virgin and short-time DBD treated sample.

**Figure 7:**
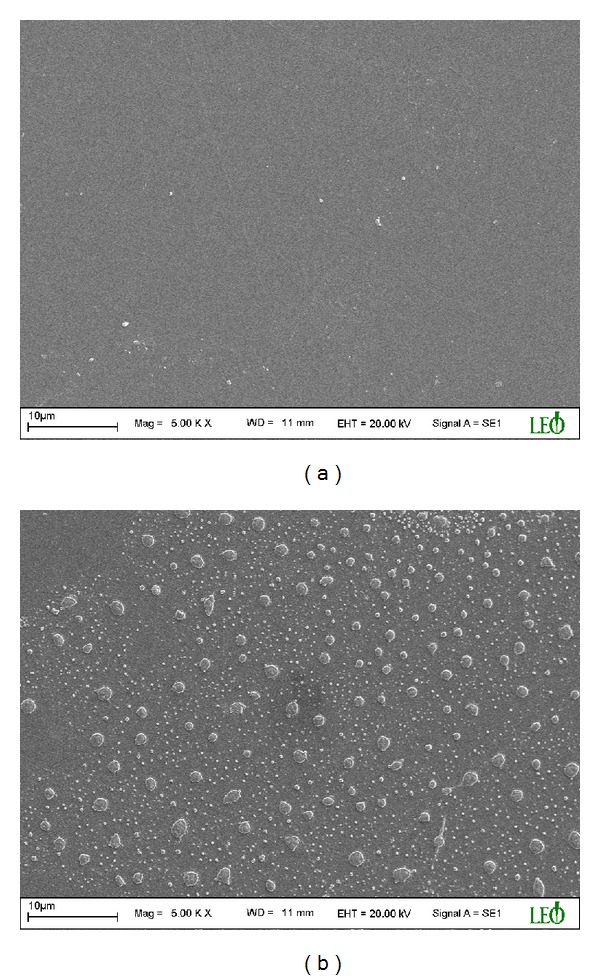
The SEM images of (a) short-time and (b) long-time DBD treated samples.

It must be noted that the level of surface functionalization and radical formation is quite low, since the DBD treatment time is low. In order to see the structural changes in the samples due to the short-time DBD effect, FTIR spectrum and SEM image of virgin and treated samples were presented in Figures 6 and 7, respectively. 

As seen in Figure 6, there are five absorbance peaks: 719 cm^−1^ and 815 cm^−1^ (CO asymmetric stretching), 1366 cm^−1^ (C−N stretching), 1710 cm^−1^ (CO symmetric stretching), and near 1778 cm^−1^ (CO asymmetric stretching). These peaks are generally accepted as characteristic of the imide rings. In Figure 6, it was observed only a very small change in the intensities of the existing absorbance peaks. Neither new formation nor an important modification was observed. Namely, it was not observed any important structural change in the sample. So the effect of surface functionalization on the adhesive properties of the sample in short-time plasma is low in comparison with the long-time plasma. In this case, the space charge effect is an important factor to analyze the changes observed in adhesive properties of the sample after the short-time DBD effect. However, if the duration of DBD effect increases, then the surface functionalization and radical formation become the most dominant factors determining the adhesive properties; therefore the effect of space charges is low in the long exposure time. The effect of short- and long-time DBD on the surface of the sample was shown in Figure 7. As seen in Figure 7, important changes and new formations were observed in long-time DBD due to the more interaction of the plasma with the sample surface.

**Figure 8:**
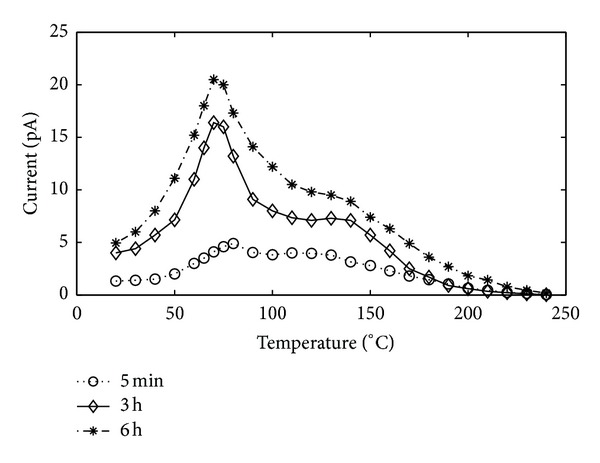
TSDC spectrums of PI exposed to DBD in SF_6_  for different treatment times.

TSDC spectrum measured for PI sample exposed to DBD treatment in SF_6_ for 5 min, 3 h, and 6 h was presented in Figure 8. There were two maximums at the temperature values of *T*_max⁡1_ = 70°C and *T*_max⁡2_ = 130°C in TSDC spectrum. As seen from the figure, the increase in DBD treatment time resulted in (i) an increase in maximum value of the amplitude, (ii) an increase in depolarization time. In other words, the increase in DBD treatment time caused an increase in the area below TSDC spectrum. As seen, the current passing through the sample decreased to a definite value with the increase of the temperature of measurement cell where the sample placed, and then it reached to a second maximum value rapidly and thereafter approached to zero. 

Now, let us consider the formation of space charges in the layers of the sample and choose the symmetric plane of electrode system as the origin of coordinate system. Since the diameters of electrodes are bigger than both the thickness of the sample and gas gap between the glass barrier and sample, it is convenient to investigate the process in one dimension. Firstly a voltage was applied to the electrode system, and then a discharge ignited in the gas gap between the sample and dielectric barrier; after a very short time, positive and negative charges settled onto the surface of the sample from the discharge gap. 

If the duration between two consecutive discharges at a definite point is long enough and the formation of discharge channels has a statistical character, then the charges settling onto dielectric surface from discharge gap have a uniform distribution character in this period due to Coulomb interaction on dielectric surface. In this case, time-dependent electrical field inside the sample will be zero because of two reasons: (i) the symmetry of the electrode system, (ii) the equality of average densities of positive and negative charges on the sample surface. But this case depends on the satisfaction of one of the following conditions: (i) both positive and negative charges have equal diffusion coefficients (ii) the charges are not able to diffuse into the sample. However, since the diffusion coefficient for electrons is much greater than that of the ions, the diffusion current created by electrons becomes more dominant. In diffusion process, the electrons, which are settled onto the surface of dielectric from discharge gap, firstly pass into the conduction band of the insulator. Then, they quickly transfer their energy to the lattice and are seized by low local energy levels. Practically, these electrons can not move into the deeper layers of the insulator. The subsequent electrons begin to fill the neighboring states, and in the course of time the most of the localized states close to the surface are occupied by diffusing electrons. On the other hand, negative charges trapped by localized energy levels while diffusing into the bulk of the sample result in a reverse electrical field. This electrical field prevents the diffusion of the new charges into the bulk from dielectric surface, and this creates conduction current in the bulk. According to mentioned phenomena, we can determine the trapped charge density by using continuity equation of the current and Poisson equation as [19]

(3) $$\begin{array}{c} n_{t}\left(x\right)=\frac{\varepsilon_{r}\varepsilon_{0}C\;}{e\left(\theta +1\right)}\text{se}{\text{c}}^{2}\left(x\cdot \sqrt{\frac{eC}{2kT_{e}}}\right);\;-L\leq x\leq +L, \end{array}$$

where *e* is the charge of the electron, *T*_*e*_ is the electron temperature, *k* is the Boltzmann constant, *θ* is the capturing parameter, and *L* is the thickness of the sample. In the expression given above, the constant value of *C* is obtained with the aid of experimental results with a method similar to one given in [3]. Numerical value of the constant *C* is calculated by the measurement of induced potential experimentally and is equal to 5 · 10^7^ V/m^2^. So we can obtain the electrical charge in unit surface area as follows [19]:

(4) $$\begin{array}{c} Q=-2\varepsilon_{r}\varepsilon_{0}\sqrt{\frac{2kT_{e}C}{e}}\cdot \tan\sqrt{L\;\cdot \;\left(\frac{eC}{2kT_{e}}\right)}. \end{array}$$

The amount of charge estimated according to this equation is greater than that determined experimentally. This situation can be explained by uniform distribution of trapped charges in the sample with time according to the symmetry of the plane (*E* = 0). In this case, the charges emerging from trapped energy levels compensate each other, so TSDC can not be observed. If the distribution form of space charges in dielectric is known, then the effective depth of the charges can be determined. 

## 4. Conclusion 

Space charge formation in PI material resulted in the improvement of adhesive properties of the material, while it degrades the insulating properties under certain conditions. The adhesive and insulating properties, which are important fundamental electro-physical properties of the material, were interrelated, so this relation can be considered in the manufacturing and exploitation of the material.

In this context, we observed that homocharges were stored in PI exposed to short-time DBD in SF_6_ medium. We suppose that the formation of space charges in material by DBD effect results from the diffusion mechanism owing to the presence of gradient of charge density in the interface of discharge channel and material. 

Equations (3) and (4) which were used to estimate the total charge and concentration of trapped charges in the sample were derived by considering the approach for the mechanism of space charge formation in the sample. The results obtained by using derived equations agreed with the experimental results obtained in the former studies [2, 3, 5, 12]. 

The relation between the surface charge density and the surface energy of the treated sample was evaluated and presented in accordance with the measurements of surface potential and contact angle. It was observed that the surface energy of DBD treated material decreased as the surface charge density of the material decreased with time. It was seen that especially for the short-time DBD the variation in surface energy due to the time can not be only ascribed to the relaxation of activated species, functional groups, and radicals. The effect of space charges on adhesive properties should be also considered for short-time DBDs. This relation is supposed to contribute to the clarification of the unclear aspects of adhesive properties and dielectric properties of polymers.
